# Team-Based Approach to Reduce Malignancies in People with Diabetes and Obesity

**DOI:** 10.1007/s11892-023-01518-y

**Published:** 2023-08-03

**Authors:** Ziyue Zhu, Samuel Yeung Shan Wong, Joseph Jao Yiu Sung, Thomas Yuen Tung Lam

**Affiliations:** 1 Stanley Ho Big Data Analytic and Research Centre, The Chinese University of Hong Kong, Shatin, Hong Kong; 2 The Jockey Club School of Public Health and Primary Care, The Chinese University of Hong Kong, Shatin, Hong Kong; 3 Lee Kong Chian School of Medicine, Nanyang Technological University, Singapore, Singapore

**Keywords:** Team-based approach, Multidisciplinary care, Metabolic syndrome screening, Cancer screening, Diabetes mellitus, Obesity

## Abstract

**Purpose of Review:**

Numerous observations have indicated an increased risk of developing various types of cancers, as well as cancer-related mortality, among patients with diabetes and obesity. The purpose of this review is to outline multiple-cancer screening among these patients through a team-based approach and to present the findings of a pioneering integrated care program designed for patients with obesity with a specific emphasis on cancer prevention.

**Recent Findings:**

A community-based multi-cancer prevention program, which provides all services in one location and utilizes team-based approaches, is reported to be feasible and has the potential to enhance the uptake rate of multiple cancers screening among patients with diabetes and obesity.

**Summary:**

The team-based approach is a commonly utilized method for managing patients with diabetes, obesity, and cancer, and has been shown to be efficacious. Nevertheless, research on team-based cancer screening programs for patients with diabetes and obesity remains limited. Providing a comprehensive screening for colorectal, prostate, and breast cancer, as well as metabolic syndrome, during a single clinic visit has been proven effective and well-received by participants.

**Supplementary Information:**

The online version contains supplementary material available at 10.1007/s11892-023-01518-y.

## Introduction

Obesity is a global epidemic that affects people of all ages, genders, and socioeconomic backgrounds [6, 79]. According to the 2021 World Health Organization (WHO) obesity and overweight fact sheets, worldwide obesity had nearly tripled since 1975, with more than 1.9 billion adults being overweight and over 650 million of these being obese. This trend of obesity poses a considerable cost burden on healthcare systems worldwide. A recent study estimates that the economic impact of overweight and obesity in 2019 is at 2.19% of global gross domestic product (GDP), and if the trends continue, the impacts are projected to rise to 3.29% of global GDP by 2060 [61]. It is well established that being overweight and obese is associated with an increased risk of various cancers, including meningioma, multiple myeloma, renal and pancreatic adenocarcinomas, hepatocellular carcinoma, gastric cardia, endometrial, esophageal, colorectal, postmenopausal breast, ovarian, gallbladder, and thyroid cancers [9, 23]. Obesity also contributes to the development of type 2 diabetes mellitus (T2DM). The expansion of adipose tissue in patients with overweight and obesity leads to an increase in the release of adipokines, which can trigger chronic inflammation and cause insulin resistance. Insulin resistance in turn results in hyperglycemia, which can eventually lead to the development of T2DM [4, 29]. The increasing prevalence of overweight and obesity has led to a corresponding rise in the rates of T2DM [21].

To reduce the risk of developing T2DM and cardiovascular disease, asymptomatic individuals are advised to undergo metabolic syndrome screening if they are overweight and have at least one risk factor, such as a first-degree family history of DM, a history of cardiovascular diseases, hypertension, dyslipidemia, physical inactivity, high-risk race or ethnicity, or other clinical conditions associated with insulin resistance [5, 34–36, 42].

DM epidemic is emerging, with more than 500 million adults living with DM across the globe [75]. The direct cost of adults with DM has been considerably increasing, with a 316% growth from 2007 to 2021 [75]. The International Diabetes Federation (IDF) estimates that the total DM-related health expenditure would reach USD 1 trillion by 2030 [75]. Patients with T2DM have an increased risk of developing both microvascular complications and macrovascular complications, including retinopathy, nephropathy, neuropathy, and cardiovascular comorbidities [26]. In addition, studies suggest that T2DM is associated with an excess relative risk of breast, endometrial, pancreatic, colorectal, and liver cancers [12, 31, 41, 49, 80]. Furthermore, there is evidence showing that T2DM is also associated with increased cancer mortality [18, 24, 66, 77]. Although the mechanism between cancer and T2DM has not been completely understood yet, genetic factors, obesity, inflammation, oxidative stress, hyperglycemia, hyperinsulinemia, cancer therapies, insulin, and certain oral hypoglycemic drugs have been identified as contributing to the associations between T2DM and cancers [82]. Despite the evidence of a positive association between cancer and T2DM, the cancer screening uptake rate is significantly lower among patients with T2DM when compared to patients without T2DM [74]. Therefore, it is essential to closely monitor patients with T2DM for the development of cancer and to implement strategies to promote cancer screening uptake.

Team-based integrated care is the provision of personalized healthcare services to accomplish shared goals within and across settings to achieve coordinated, high-quality care by at least two healthcare providers from different disciplines who work collaboratively with patients and their caregivers [54]. Team-based integrated care has been utilized to manage patients with T2DM to reduce their risk of cardiovascular and renal complications, hospitalization, and mortality [59]. As the fact that T2DM is associated with multiple cancers, a team-based approach is also appropriate to promote cancer screening and reduce cancer risk among patients with T2DM [59]. Additionally, obesity is not only a risk factor for T2DM, but also a major barrier to accessing cancer screening services [33], and it is also an important confounder in the association between T2DM and malignancies [50]. It is suggested that obesity and T2DM share the same mechanisms of increasing cancer risk through cellular proliferation, inflammation, and hormonal balance [15, 82]. To prevent malignancies in patients with T2DM and obesity, cancer screening should be tailored using the team-based approach for these high-risk populations to promote primary prevention and early detection.

This review aims to provide an overview of the team-based approach for obesity, T2DM, and cancer screening. Additionally, we will present the organization and results of a screening program that targets patients with overweight and obesity, which combines metabolic syndrome screening and multi-cancer screening in a one-stop way using a team-based approach.

## Search Strategy and Results

The search strategy included a logical combination of keywords and standardized medical subject headings terms in medical literature databases. Keywords such as “team-based approach,” “multidisciplinary,” “integrative team,” “interdisciplinary,” “whole team approach,” “interprofessional team,” “healthcare team,” “malignancies,” “cancer,” “carcinoma,” “tumour,” “diabetes mellitus,” “DM,” “diabatic,” “diabetes,” “DM management,” “obesity,” “overweight,” “high BMI,” and “unhealthy weight” were used to identify journal articles that address the utilization of a multidisciplinary approach to reduce cancer risk in patients who are obese with T2DM. A thorough search across various databases, including PubMed, EMBASE, Medline, Cochrane, and CINAHL Ultimate was conducted. Additionally, the references and related articles of the included studies were also manually examined. There is no literature on cancer screening for patients with both obesity and T2DM. Hence, this review focuses on summarizing the team-based approach of cancer screening, obesity, and diabetes management separately and offering suggestions on how to establish and manage a multidisciplinary team to address the cancer screening issue for patients with obesity and T2DM.

## The Team-Based Approach to Obesity Management

It is well-established that obesity is one of the major risk factors for T2DM. Additionally, there is also increasing evidence that obesity increases the risk of developing and dying from malignancy [15, 16, 40]. The American Society of Clinical Oncology (ASCO) launched an Obesity Initiative in 2013 and promoted multidisciplinary collaboration in healthcare provider education and training; public education and activation; research; and policy and advocacy, so as to reduce cancer incidence among patients with obesity [51]. According to the guideline of the American College of Cardiology (ACC)/American Heart Association (AHA)/The Obesity Society (TOS), the treatments for overweight and obesity are eating plan improvement and regular physical activity, behavioral intervention, weight-management programs, weight-loss medications, weight-loss devices, bariatric surgery, and special diets [44]. In relation to the team-based management for patients with obesity, enrolling them into a multidisciplinary weight loss program is one of the most efficient methods and also helps patients maintain long-term weight loss [55].

A multidisciplinary weight management team should consist of obesity medicine physicians, dietitians, physical trainers, psychologists or behavioral therapists, and bariatric surgeons [37]. A randomized control trial conducted in Germany from 2014 to 2020 demonstrates that a medically supervised high-intensity program comprising meal replacements, behavioral education, and a supervised physical exercise program leads to clinically significantly greater weight loss at 26 and 52 weeks compared with invasive intragastric balloon therapy for patients with obesity [62]. Another example in Australia, the “Healthy Weight Clinic,” shows that multidisciplinary obesity service enables patients to achieve clinically meaningful weight loss and improve skeletal muscle mass to body fat ratio with the weight loss maintained at least 1 year post-intervention [20]. An ongoing randomized control trial, TECNOB (TEChnology for Obesity), presents interim results of a multidisciplinary telecare intervention for obese patients with T2DM. The researchers found that information and communication technologies can help clinicians to deliver treatment in a cost-effective and time-saving manner to a large number of obese individuals with comorbidities [17]. If other non-invasive weight-loss interventions fail after 6 months, bariatric surgery is an established and effective part of weight-loss management for severely obese patients [53]. Furthermore, complementary and alternative therapies have also been suggested to have encouraging effects on the treatment of obesity [25, 53].

As obesity is an increasingly significant health problem among children and adolescents [43], special care should be paid to overweight children and adolescents to prevent the development of serious complications such as T2DM and cardiovascular diseases [13, 79]. The team-based approach can also be applied in this context. An American study aimed to prevent T2DM among Latin adolescents with obesity through a community-based program has proved a success in improving cardiometabolic and psychosocial health [71•]. Among all members of the multidisciplinary team, nurses are in a unique position across healthcare and community-based settings as they interact with families and play a key role in the prevention and management of overweight and obesity in children and adolescents [64]. Adapted lifestyle intervention programs should be tailored and applied to other vulnerable populations such as pregnant women and elder individuals [60], and the corresponding research projects should track their clinical outcomes and cost-effectiveness.

## The Team-Based Approach to Diabetes Management

T2DM is a complicated chronic disease that requires the expertise of a diverse team of medical specialists who should master different facets of T2DM treatment, including the clinical evaluation of complications and comorbidities. Some of the most common complications of T2DM include coronary heart disease, stroke, peripheral arterial disease, diabetic kidney disease, retinopathy, and peripheral neuropathy [76]. Corresponding to these complications, an interdisciplinary team should involve endocrinologists, radiologists, cardiologists, vascular surgeons, neurologists or neurosurgeons, nephrologists, and ophthalmologists. Apart from these well-documented complications, there are emerging complications such as cognitive disability and affective disorders which requires psychologists and psychiatrists included in the team [38, 76]. In addition to the clinical treatment, the latest American Association of Clinical Endocrinology Clinical Practice Guideline (AACE) in 2022 also advocates lifestyle interventions for optimal glycemic control [14]. The multidisciplinary behavior intervention team is advised to include combinations of dietitians, nurses, health educators, physical trainers, and clinical psychologists [14].

Numerous studies have examined the pathophysiology of various complications in patients with T2DM and their multidisciplinary implications. Cardiovascular disease is one of the leading causes of death among patients with T2DM [65], and patients with T2DM are nearly twice likely to experience a heart attack or stroke than those without T2DM [10, 11]. Hence, some researchers suggest that a cardiologist and an endocrinologist should provide joint supervision of the patients based on a protocol that ensures effective communication and exchange of information, rather than working in parallel [2, 7, 68]. Recent evidence from research on a patient-centered, team-based intervention, “Center for Integrated and Novel Approaches in Vascular-Metabolic Disease (CINEMA),” shows that a 1-year intervention improves control of multiple cardiovascular risk factors, with patients with T2DM significantly reduced glycosylated hemoglobin, total cholesterol, low-density lipoprotein cholesterol, systolic blood pressure, and body mass index [58]. Additionally, as one of the primary causes of chronic kidney disease (CKD), the rise in T2DM prevalence also contributes to the emergence of CKD [47]. A randomized clinical trial integrating the Joint Asia Diabetes Evaluation (JADE) web portal, nurse reminders, and team-based care for patients with diabetic kidney disease (DKD) demonstrates that patients in a web-assisted team-based powered group are not only more likely to achieve multiple treatment targets, such as HbA1c, blood pressure, and low-density lipoprotein cholesterol level, but also have a lower incidence of cardiovascular, kidney, and cancer events [19]. Apart from web-based management, another technology-assisted team-based approach, telemedicine that involves an interpersonal and telemedicine-based exchange of hospital routine data between specially trained diabetes nurses and endocrinologist also improves efficacy, safety, and efficiency of diabetes care in inpatient settings [67].

Another common complication in patients with T2DM is the diabetic foot, and the pathology is linked to peripheral vascular disease and diabetic neuropathy. It is estimated that the lifetime risk of developing a diabetic foot ulcer in patients with DM is 19 to 34% [28]. Research indicates that the presence of a multidisciplinary foot care team, in conjunction with evidence-based prevention and management, can decrease the occurrence of lower extremity amputation related to diabetes. In order to form a multidisciplinary team, it is necessary to bring together experts from different fields including endocrinology, surgery, radiology, infectious diseases, nursing, podiatry, and orthotics [22, 48•]. Oral manifestations belong to another cluster of complications for T2DM. Proper brushing and flossing behaviors, encouraging patients to visit the dentist for a routine check-up, and controlling blood glucose levels would prevent oral manifestations [57]. However, unlike diabetic foot ulcers, patients with T2DM have poor knowledge and awareness of the association between T2DM and oral health [3]. A “CODAPT-My Care” pathway connecting dental clinics with primary care discusses that members in the expert panel should comprise family medicine physicians, periodontists, endocrinologists, and clinical pharmacists [1]. An example of the prevention program, the Lifestyle Change plus Dental Care (LCDC), pays special attention to lifestyle changes and periodontal care to avoid dental complications [69]. As for diabetic-related retinopathy screening, teleophthalmology is an effective method for encouraging patient engagement [73]. In a community-based teleophthalmology program, the primary care physicians and nurses are responsible for screening education, managing the records, outreaching patients with DM, and having their retinal images taken which are sent to remote ophthalmologists electronically to make diagnoses [52]. The team-based practices for diabetic complications are not exhaustively listed here.

There remains a main issue that the team-based approach is hard to replicate in the community; efforts have been made to develop protocols and guidelines that can facilitate the implementation of interdisciplinary team care of patients with T2DM in community settings. A recently published study design called the Diabetes Complication Control in Community Clinics (D4C) study is a cluster-randomized trial conducted among 38 community health centers in China [70]. In addition to the members usually included in DM management, such as endocrinologists, cardiologists, and primary care physicians, the study design proposes to add a clinical decision support system that integrates guideline-based treatment algorithms for glycemic, blood pressure, and lipid control, together with a patient’s medical history and insurance policy [70]. Although the results of the D4C trial have not been published yet, it presents a cluster-randomized trial design that provides a roadmap for team-based primary care in community clinics in Asia.

## Team-Based Approach for Cancer Screening in Patients with Diabetes

Identifying high-risk patients is critical in cancer screening, so as to detect cancers of early stage and precursor lesions, hence improving survival rate if intervened at an early stage [8, 30, 32]. Additionally, as rates of cardiovascular mortality among patients with T2DM decline, cancer mortality now accounts for a larger proportion of deaths and cancer is an emerging cause of death in some countries [39, 63, 76]. Although patients with T2DM and obesity are known as high-risk populations for several cancers, based on the literature search conducted in this review, the literature on cancer screening programs tailored for them is limited. In the following sections, a framework for cancer screening in patients with T2DM will be proposed.

### The Task of a Multidisciplinary Team

Team-based approach or multidisciplinary approach is defined as providing health services through at least two health providers that work collaboratively. Generally, a multidisciplinary team should consist of at least one representative who cares for patients, and all team members meet regularly to make consensus diagnostic and management recommendations for patients. The main tasks of a multidisciplinary clinic for patients with T2DM are the treatment of existing T2DM, the prevention and treatment of complications, and the screening of cancers.

There is limited prevention practice to support the effectiveness of a multidisciplinary cancer screening team in reducing the risk of cancers among patients with T2DM. Systematic knowledge of the pathophysiology of various cancers in patients with T2DM is largely lacking, and the gaps require further discoveries. Additionally, the prevalence of specific cancer varies across countries, leading to a significant variation in the composition of interdisciplinary teams. Therefore, the optimal composition of such teams should be dependent on local experiences. A general discussion on the multidisciplinary team organization would be provided in this review.

### Multidisciplinary Team Size

Referring to an overview of strategies for implementing multidisciplinary teamwork in cancer healthcare [72], a multidisciplinary team for a certain disease requires a core treatment board, a chair of the team, nurses, coordinators, and other administrative members. Following the discussions of the multidisciplinary team building in general cancer care [72], hepatocellular carcinoma [56], and diabetic foot ulcers [48•], hereby this review proposes a multi-level multidisciplinary structure in Table 1. The first level focuses on the treatment of T2DM which involves general practitioners, endocrinologists, and diabetic nurses. The second level would focus on the management of complications of T2DM. As discussed in the former section, the team should include radiologists, cardiologists, vascular surgeons, neurologists or neurosurgeons, nephrologists, ophthalmologists, psychologists, and psychiatrists. Most diabetes clinics have multidisciplinary structures for the first two levels, but to our knowledge, there are no diabetes clinics that offer cancer screening in addition to regular T2DM therapy. Hence, level 3 is introduced in the framework which entails cancer screening specialists. For example, patients with T2DM have a higher risk of colorectal and breast cancer [49, 80], and including gastroenterologists and radiologists would reduce patients’ cancer risk at an early stage. As recommended in AACE guideline [14], lifestyle intervention team members and non-clinical members such as registered dietitians, physical trainers, health educators, social workers, marriage and family therapists, and behavioral therapists are advised to be included in level 4. Additionally, new communication technologies, such as teleconferencing or teleconsultation, which offer the possibility of expansion into underserved or rural areas, as well as areas such as correctional facilities, should be included in the multidisciplinary team [56]. On top of this multi-level framework, administrative support is another important factor for implementing a team-based approach successfully [72]. Nurses should also be a part of the team to administer and coordinate the program, recruit patients into the program, maintain patients’ data, track assessments, and ensure follow-up visits [46].

**Table 1:** Multi-level multidisciplinary framework

Level of care	Specialists involved
Level 1	General practitioners, endocrinologists, and diabetic nurses
Level 2	Level 1 and complication practitioners, such as radiologists, cardiologists, vascular surgeons, neurologists or neurosurgeons, nephrologists, ophthalmologists, psychologists, and psychiatrists
Level 3	Level 2 and specialists working together in a multidisciplinary way with special expertise in cancer screening
Level 4	Level 3 and lifestyle intervention team members and non-clinical members such as registered dietitians, physical trainers, health educators, social workers, marriage and family therapists, and behavioral therapists

Before implementing the multidisciplinary team, a strategic plan that includes the aim and vision of the team, the endpoint definition and the evaluation, the components of the team, and the analysis of benefits and costs should be made [56]. During the execution of the multidisciplinary screening procedure, team members should meet at periodic intervals (such as fortnight or bimonthly) and on demand to discuss, diagnose, and reach a consensus regarding patients’ complex conditions.

### Advantages of the Multidisciplinary Team

Multidisciplinary teamwork has the features of coordination throughout treatment plan development, streamlined treatment pathways, and reduced duplication of services, resulting in increased survival rates, better treatments, and easier access to information for patients with T2DM [1, 2, 7, 37, 78]. Unfortunately, the clinical link between a team-based approach and the reduction of cancer risk has not been established; hence, further research is needed. An ongoing study on the team-based approach in cancer screening for asymptomatic patients with overweight and obesity is described below as an example.

## An Example of a Team-Based Approach to Reducing Malignancies in Asymptomatic Individuals with Overweight or Obesity

### Study Setting

A charity-sponsored, one-stop, community-based, multi-cancer and metabolic syndrome screening program, under the auspice of the Faculty of Medicine at the Chinese University of Hong Kong, was launched in 2018. This is a 6.5-year program, run by a multidiscipline, including research nurses, primary care physicians, gastroenterologists, general surgeons, urologists, radiologists, radiographers, breast surgeons, endocrinologists, medical social workers, dietitians, and physical trainers. The multidisciplinary team has regular monthly meetings to review and discuss the logistics and performance of this integrated screening program. During the COVID pandemic, monthly meetings continued via teleconference. A more detailed study setting is well-described elsewhere [45••].

### Study Population

Asymptomatic and screening naïve individuals of ages 50 to 75 were recruited across the territories via media promotion and advertisement. Subjects who are overweight (BMI 23–24.9 kg/m^2^) or obese (BMI ≥ 25 kg/m^2^) are a higher priority to participate in this program because of their increased risk of colorectal, prostate, and breast cancer [16, 40, 81]. Subjects who have a strong family history of colorectal cancer (CRC) (two or more first-degree relatives diagnosed CRC), personal history of colonic adenoma, diverticular disease, inflammatory bowel disease, prosthetic heart valve or vascular graft surgery, or medical conditions which are contraindications for colonoscopy are excluded from CRC screening. Subjects who have a personal history of prostate cancer or significant medical conditions that may result in limited life expectancy (<10 years) are excluded from prostate cancer screening. Subjects who have a personal history of breast cancer are excluded from breast cancer screening.

### Multiple Cancers Screening

Screening interval and screening strategies of initial and subsequent tests for colorectal, prostate, and breast cancers were based on recommendations from various international and local guidelines (supplementary table 1). Biennially colorectal cancer, prostate cancer (for male subjects), and breast cancer (for female subjects) screening are arranged in one go during the initial visit. All eligible subjects are offered fecal immunochemical tests (FIT) by primary care physicians for CRC screening. Subjects with positive FIT are referred to gastroenterologists or general surgeons for colonoscopy. After acceptance of CRC screening, male subjects are invited to receive prostate cancer screening by taking blood for prostate antigen (PSA) and prostate health index (PHI) tests by research nurses for prostate cancer screening. Subjects with positive PSA/PHI (PSA > 10 ng/mL or PHI ≥ 35, for PSA between 4 and 10 ng/mL) are referred to urologists for ultrasound-guided prostate biopsy. After acceptance of CRC screening, female subjects are invited to receive mammography by radiographers for breast cancer screening. If the mammography is positive (those with Breast Imaging-Reporting and Data System [BI-RADS] category ≥4), subjects are referred to breast surgeons for ultrasound-guided breast biopsy. For those who have negative FIT, PSA/PHI, or mammography results, research nurses explain the results and instruct them to repeat colorectal, prostate, or breast cancer screening after 2 years, respectively. Any subjects who are diagnosed with cancer and need emotional or financial support are referred to medical social workers (supplementary figure 1). The primary outcome is the feasibility and acceptability of one-stop team-based multi-cancer screening, i.e., the acceptance rate for a second cancer screening (prostate cancer for males and breast cancer for females) after accepting CRC screening.

### Metabolic Syndrome Screening

Apart from the screenings for CRC, prostate cancer, and breast cancer, recruited subjects are also offered to screen for metabolic syndrome. Measurements of body height, weight, body mass index (BMI), waist circumference, and blood pressure are performed during the initial visit. The blood sample is taken for fasting glucose and lipid profile tests. Subjects who have fasting blood glucose ≥7 mmol/L are referred to endocrinologists for comprehensive DM assessment. Those who are found to be pre-diabetes (fasting blood glucose 6.1–6.9 mmol/L) are educated by research nurses about healthy lifestyle modifications and advised to recheck in 1–2 years. Ten-year atherosclerotic cardiovascular disease (ASCVD) risk is calculated based on fasting lipids profile results, blood pressure, medical history, and demographics. Those who have ASCVD risk ≥7.5% and <15% are educated by research nurses for healthy lifestyle modifications and advised to recheck in 1–2 years, while those ≥15% are referred to dietitians and physical trainers for significant lifestyle modifications, such as dietary change and physical activity and referred to primary care physicians for further management (supplementary figure 1).

### Interim Results

#### Baseline Characteristics

From August 2018 to February 2023, a total of 7377 (mean age 59.7 ± 5.4, male 47.8%) were recruited. Among them, 1426 (19.3%) subjects had DM while 1542 (20.9%) subjects were overweight and 4573 (62.0%) were obese. A total of 6870 (93.1%) subjects were eligible for colorectal cancer screening, while 3404 (46.1%) and 3345 (45.3%) subjects were eligible for prostate and breast cancer screening, respectively. Other baseline characteristics are listed in Table 2.

**Table 2:** Baseline characteristics of 7377 asymptomatic subjects

Total number of subjects	7377
Number of subjects eligible for colorectal cancer screening (%)	6870 (93.1)
Number of subjects eligible for prostate cancer screening (%)	3404/3526 (96.5)
Number of subjects eligible for breast cancer screening (%)	3345/3851 (86.9)
Male gender (%)	3526 (47.8)
Mean age (SD)	59.7 (5.4)
Overweight (≥23 and <25 kg/m^2^) (%)	1542 (20.9)
Obesity (≥25 kg/m^2^) (%)	4573 (62)
Central obesity (waist circumference ≥90 cm for male and ≥80 cm for female) (%)	5902 (80)
Smoking, current or past (%)	917 (12.4)
Alcohol drinking (%)	778 (10.6)
First degree family history of colorectal cancer (%)	674/6870 (9.8)
First degree family history of prostate cancer (%)	234/3404 (6.9)
First degree family history of breast cancer (%)	552/3345 (16.5)
Diabetes (%)	1542 (20.9)
Hypertension (%)	2510 (34)
Ischemic heart disease (%)	85 (1.2)
Chronic obstructive pulmonary disease (%)	99 (1.3)
Stroke (%)	105 (1.4)
Fatty liver (%)	905 (12.3)
Cirrhosis (%)	9 (0.1)
Gastroesophageal reflux disease (%)	616 (8.4)
Married (%)	5744 (77.9)
Secondary education level or above (%)	6402 (86.8)
Full-time employment (%)	3082 (41.8)
Personal monthly income above median (≥$20,000) (%)	
Residential area	
Hong Kong Island (%)	1038 (14.1)
Kowloon (%)	2045 (27.7)
New Territories (%)	4192 (56.8)
Islands (%)	102 (1.4)

#### Multiple Cancers Screening Results

**Fig. 1:**
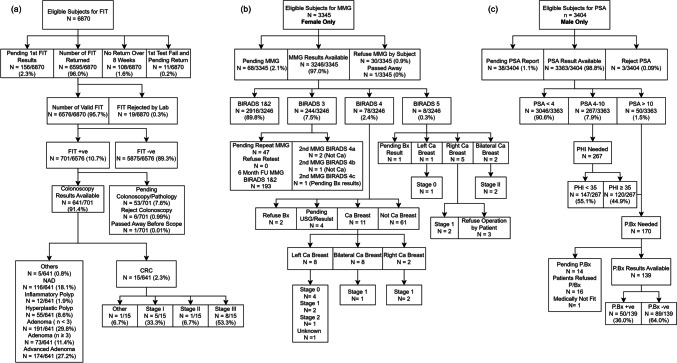
Multi-cancer screening results. **a** Colorectal cancer. **b** Breast cancer. **c** Prostate cancer

In this one-stop multi-cancer screening study, after accepting CRC screening, the acceptance rates for a second cancer screening (prostate cancer for males and breast cancer for females) were 99.9% and 99.1%, respectively. Screening results of CRC, breast, and prostate cancer were illustrated in Fig. 1a, b, and c, respectively. Briefly, the positivity rate of FIT, PSA/PHI, and mammography were 701/6576 (10.7%), 170/3363 (5.1%), and 86/3245 (2.7%). After referring to colonoscopy, prostate, and breast biopsy, 15/641 (2.3%), 50/139 (36%), and 19/80 (23.8%) subjects were diagnosed with CRC, breast, and prostate cancer, respectively. For CRC screening, there were 438/641 (68.3%) subjects diagnosed with pre-cancerous lesions (advanced adenoma and adenoma).

#### Metabolic Syndrome Screening Results

One hundred sixteen (1.6%) subjects were found to have newly diagnosed T2DM after comprehensive DM assessment because of elevated fasting glucose (≥7 mmol/L). Another 1815 (24.6%) subjects were found to be pre-diabetes (6.1–6.9 mmol/L). A total of 1951 (26.4%) subjects were found to have ASCVD risk 15% while 1909 (25.9%) had ASCVD risk ≥7.5% and <15%.

### Strengths of Team-Based Approach One-Stop Multi-cancer and Metabolic Syndrome Screening

The one-stop multi-cancer screening program is one of the pioneers of integrated programs that target cancers and metabolic syndrome screening in a population with overweight and obesity. Recruiting participants and participants’ compliance are major difficulties in cancer screening research. Three important factors for high acceptance of multiple-cancer screening are identified in this program: the provision of knowledge of cancers, counseling for individual patients, and free-of-charge services. The interim results show that the acceptability of one-stop team-based multi-cancer screening is very high (>99%) which in turn promotes the uptake of various cancers screening [45••]. The periodic meeting requirement for the multidisciplinary team would ensure the frequency of communications that benefit the patients with better diagnoses [45••]. From the logistic perspective, different specialists and researchers would have access to a compact, well-organized, and longitudinal database that tracks a large prospective cohort over time. The database would work as the fundamental for further clinical research. Furthermore, the program provides an advanced experience for making health public policies.

### Limitations of Team-Based Approach One-Stop Multi-cancer and Metabolic Syndrome Screening

Although the feasibility and effectiveness of the team-based one-stop program are well-documented, the cost-effectiveness has not yet been examined. Additionally, a subgroup of a relatively more health-conscious asymptomatic population aged 50 to 75 who are self-referred to the program was selected; hence, the screening results may not be generalizable. Furthermore, the study is not population-based but a community-based design involving a single center. However, as participants are recruited across the territories, this screening cohort can largely represent the general population of Hong Kong because they are in proportion the same as the distribution of the Hong Kong population by age group, sex, and residential district [27, 45••]. Lastly, there is a hierarchical design in this study that individuals are introduced for CRC screening first, then are asked for taking prostate cancer screening for males and breast cancer screening for females. The individuals who are merely eligible or willing to undergo prostate or breast cancer screening are unobserved.

## Conclusions

Multidisciplinary teamwork promotes exchanges of information between specialists to optimize efficiency and expedites in diagnostic capabilities, evaluation, and treatment. Existing shreds of evidence suggest that a multidisciplinary approach is able to improve diabetic and/or obese patients’ healthcare. An integrated model of cancer screening and metabolic syndrome screening is feasible and can increase the cancer screening uptake rate.

### Supplementary information

ESM 1 (JPEG 1255 kb)

ESM 2 (DOCX 19 kb)
