# Supplementary figures and images for: Team-Based Approach to Reduce Malignancies in People with Diabetes and Obesity
Source: Curr Diab Rep. 2023 Aug 3;23(10):253–63. doi: 10.1007/s11892-023-01518-y (PMC10520129; doi:10.1007/s11892-023-01518-y)

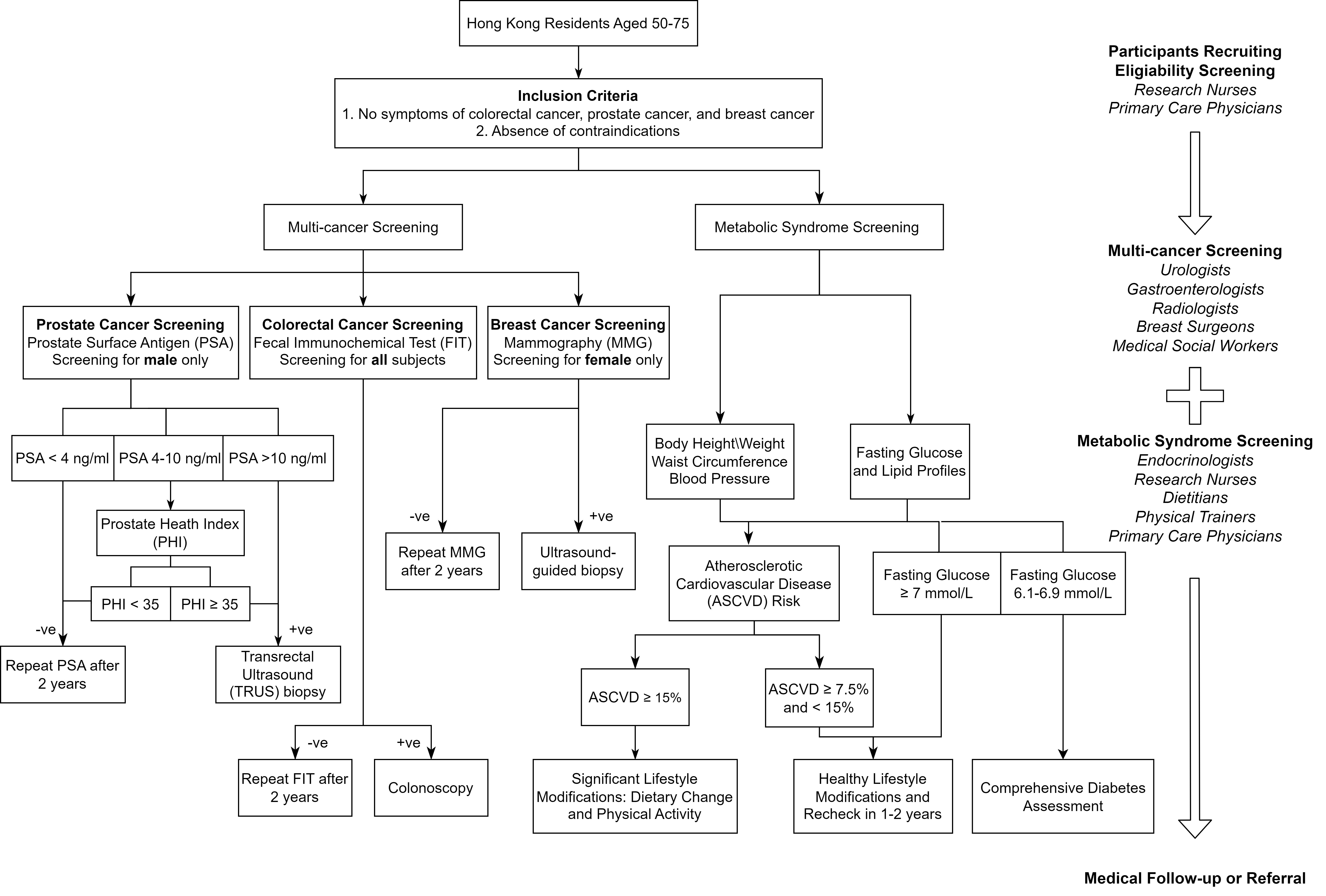

Supplement: Supplementary file 1 — (JPEG 1255 kb) [file 11892_2023_1518_MOESM1_ESM.jpeg]
