# Supplementary material for: Team-Based Approach to Reduce Malignancies in People with Diabetes and Obesity
Source: Curr Diab Rep. 2023 Aug 3;23(10):253–63. doi: 10.1007/s11892-023-01518-y (PMC10520129; doi:10.1007/s11892-023-01518-y)
Supplement: Supplementary file 2 — (DOCX 19 kb) [file 11892_2023_1518_MOESM2_ESM.docx]

Supplementary table 1. Summary of international and local guidelines for screening of colorectal, prostate and breast cancer.

|  | **Recommended age of screening** | **Screening tests and screening interval** | **Subsequent tests if initial screening test positive** | **Guidelines** |
| --- | --- | --- | --- | --- |
| **1. Colorectal cancer** | 50-75 years | Faecal immunochemical tests (FIT) 1-2 yearly | Colonoscopy | The Asia Pacific consensus statements [a];  The US Preventive Services Task Force [b]; The American Cancer Society, the US Multi-Society Task Force on Colorectal Cancer, the American College of Radiology [c];  Cancer expert working group on cancer prevention and screening [d] |
| **2. Prostate cancer** | (i). 55-77 years | (i). Prostate Specific Antigen (PSA) 2-yearly | (i). Trans-rectal ultrasound and prostate biopsy | (i). Position Statements on the use of PSA for early detection of prostate cancer jointly issued by the Hong Kong Urological Association and the Urological Board of College of Surgeons of Hong Kong [e] |
|  | (ii). 55-69 years | (ii). Prostate Specific Antigen (PSA) 2-yearly | (ii). Trans-rectal ultrasound and prostate biopsy | (ii). The US Preventive Services Task Force 2017 [f] |
|  | (iii). >50 years (Well-informed man with a good performance status and at least 10-15 years of life expectancy) | (iii). Prostate Specific Antigen (PSA) 2-yearly | (iii). Trans-rectal ultrasound and prostate biopsy | (iii). EAU Guidelines Prostate Cancer 2016 - European Association of Urology [g]  American Cancer Society [h]  Chinese Urological Association [i] |
| **3. Breast cancer** | 50-74 years | Mammography 2-3 yearly | Based on Breast Imaging-Reporting and Data System (BI-RADS) assessment category [1-6]  For BI-RAD 4 or above, perform core needle biopsy or, alternatively, surgical excision if unable to perform core needle biopsy | United States Preventive Services Task Force (USPSTF) [j]; Canadian Task Force on Preventive Health Care (CTFPHC) [k]  This breast cancer screening service was further encouraged by the American College of Physicians (ACP) as a high value preventive service. |

References

[a]:  Sung JJY, Ng SC, Chan FK, et al. An updated Asia Pacific Consensus Recommendations on colorectal cancer screening. Gut 2015; 64:121-32.

[b]: Lin JS, Piper  MA, Perdue  LA, et al.  Screening for colorectal cancer: updated evidence report and systematic review for the US Preventive Services Task Force. JAMA 2016;315:3576-94.

[c]: Levin B, Lieberman DA, McFarland B, et al*.* Screening and surveillance for the early detection of colorectal cancer and adenomatous polyps, 2008: a joint guideline from the American Cancer Society, the US Multi-Society Task Force on Colorectal Cancer, and the American College of Radiology. *Gastroenterology* 2008;134:1570–95.

[d]: Hong Kong Urological Association and the Urological Board of College of Surgeons of Hong Kong Available at: <http://www.colonscreen.gov.hk/en/public/about_crc/recommendation_of_the_cancer_expert_working_group_on_cancer_prevention_and_screening.html>. Accessed on 19 April 2017

[e]: Available at: [www.hkua.org/download/PSAtesting.pdf](http://www.hkua.org/download/PSAtesting.pdf). Accessed on 19 April 2017

[f]: Bibbins-Domingo K, Grossman DC, Curry SJ. The US Preventive Services Task Force 2017 Draft Recommendation Statement on Screening for Prostate Cancer. JAMA published online April 11, 2017. Doi: 10.1001/jama.2017.4413

[g]. Available at: <https://uroweb.org/wp-content/uploads/EAU-Guidelines-Prostate-Cancer-2016.pdf> . Accessed on 19 April 2017

[h]. American Cancer Society Recommendations for Prostate Cancer Early Detection. Available at: 2https://www.cancer.org/cancer/prostate-cancer/early-detection/acs-recommendations.html. Accessed on 20 April 2017

[i]: Chinese Urological Association. Available at: <http://www.cuan.cn/>. Accessed on 20 April 2017.

[j]. [Siu AL](https://www.ncbi.nlm.nih.gov/pubmed/?term=Siu%20AL%5BAuthor%5D&cauthor=true&cauthor_uid=26757170); [U.S. Preventive Services Task Force](https://www.ncbi.nlm.nih.gov/pubmed/?term=U.S.%20Preventive%20Services%20Task%20Force%5BCorporate%20Author%5D). Screening for Breast Cancer: U.S. Preventive Services Task Force Recommendation Statement. Ann Intern Med. 2016 Feb 16;164(4):279-96.

[k]. The Canadian Task Force on Preventive Health Care. Recommendations on screening for breast cancer in average-risk women aged 40–74 years. CMAJ November 22, 2011 vol. 183 no. 17 doi: 10.1503/cmaj.110334
